# The Effect of Antenatal Education on Expectant Fathers’ Attitudes toward Breastfeeding and Attachment to the Fetus

**DOI:** 10.3390/nursrep13010023

**Published:** 2023-02-12

**Authors:** Calliope Dagla, Evangelia Antoniou, Antigoni Sarantaki, Maria Iliadou, Irina Mrvoljak-Theodoropoulou, Ewa Andersson, Maria Dagla

**Affiliations:** 1 Department of Midwifery, School of Health & Care Sciences, University of West Attica, 12243 Athens, Greece; 2 Department of Psychology, National & Kapodistrian University of Greece, 15772 Athens, Greece; 3 Department of Women’s and Children’s Health, Division of Reproductive Health, Karolinska Institutet, 17177 Stockholm, Sweden

**Keywords:** antenatal education, expectant father, breastfeeding, attachment, fetus

## Abstract

Background: This study explores the effect of antenatal education on fathers’ attitudes toward: (i) breastfeeding and (ii) attachment to the fetus. A secondary aim is to explore the relationship of fathers’ demographic and the psycho-emotional characteristics that come with breastfeeding and attachment. Methods: This is a longitudinal study involving a group of 216 Greek expectant fathers who participated with their partners in an antenatal educational program performed by midwives in Athens, Greece (September 2020–November 2021). The Iowa Infant Feeding Attitudes Scale (IIFAS) and Paternal Antenatal Attachment Scale (PAAS) were administered at two time points: (a) 24th–28th gestation week and (b) 34th–38th gestation week. The T-test and Univariate Analyses of Variance (ANOVA) were performed. Results: The expectant fathers’ scores show that breastfeeding intention/exclusivity and prenatal attachment to the fetus were higher after their participation in the antenatal education program, but the difference was not statistically insignificant. Expectant fathers with a cohabitation agreement (*p* = 0.026), who felt very much supported by their partners (*p* = 0.001) and had no relationship difficulties with their partners (*p* < 0.001), as well as those who reported being very happy during pregnancy (*p* < 0.001), showed greater paternal antenatal attachment to the fetus. Conclusions: Although the difference was statistically insignificant, antenatal education appears to have an impact on paternal breastfeeding attitudes and antenatal attachment to the fetus. Additionally, several paternal characteristics were associated with greater antenatal attachment. Future research should be directed toward the investigation of additional factors that impact antenatal–paternal attachment and breastfeeding attitudes so that effective education programs can be designed.

## 1. Introduction

The transition to parenthood is a major life change and can be confusing and overwhelming. Parents often report physical and emotional changes after childbirth [1]. Antenatal education can play an important role in empowering, supporting, and helping new parents cope with these changes. The goals of antenatal classes may vary, but, globally, the common goal of antenatal education is to prepare new parents for childbirth, breastfeeding, and parenthood [2]. Antenatal education aims to guide pregnant women and their partners to adopt healthy behaviors, prepare them for the impending birth, and strengthen the self-confidence of new parents. Although numerous studies have been conducted on the effects of antenatal education, the evidence remains insufficient [3].

Several studies have shown that antenatal education reduces anxiety and fear during childbirth [4,5,6,7]. Additionally, antenatal education has been shown to have a positive effect on the incidence of breastfeeding [8,9,10]. According to a Spanish study, the risk of stopping breastfeeding during the first month after delivery was three times more likely for women who did not attend education classes compared to those who attended more than five classes [11]. Regarding the short-term continuation of breastfeeding, it is generally believed that antenatal education provides information and strategies to help manage the most common difficulties in the first few weeks postpartum (injured nipples, overload, etc.) [12,13,14]. In addition to childbirth and breastfeeding, studies show that antenatal education increases maternal attachment [15,16,17].

Although the benefits of antenatal education have been studied to some extent, research examining its effect on expectant fathers is limited. Historically, pregnancy and childbirth have been a predominantly female affair [18,19], with limited reports of men being involved in pregnancy or being present during childbirth [20]. However, evidence shows that most women want their partner to be involved in the pregnancy, to be present at the birth of their child, and to participate in raising their children [21,22]. Many men have also been shown to desire such involvement, but it is evident that very little or no help at all is offered to the majority of men regarding parenting. Maternal health services often exclude men and their needs as parents, although evidence shows that their involvement can positively influence the health outcomes for themselves, their partners, and their children [23]. Involvement of partners is important when it comes to breastfeeding as well. The presence of fathers can play a very important role in breastfeeding success and several studies conclude that men’s participation in antenatal breastfeeding education increases their knowledge about breastfeeding [24,25,26] and results in increased exclusive breastfeeding rates for their partners [27]. Additionally, one study has shown a correlation between antenatal education and fathers’ antenatal attachment to the fetus [28]. These findings highlight the need for more health professionals to include prospective fathers in antenatal education [29,30,31].

As highlighted above, antenatal education can offer significant benefits during the transition to the parental role. It can have an impact on pregnancy outcome, childbirth, breastfeeding, parental role, partner relationship, attachment to the fetus, and, consequently, the newborn and family functioning in general [32]. Furthermore, the growing interest in the role of the partner highlights the need to study the ways in which the partner’s involvement can be increased and their role enhanced. The aim of this study is to explore the effect of antenatal education on expectant fathers’ attitudes toward: (i) breastfeeding and (ii) attachment to the fetus. A secondary aim is to explore the relationship between expectant fathers’ demographic and the psycho-emotional characteristics that come with breastfeeding and antenatal attachment.

## 2. Materials and Methods

### 2.1. Procedure

#### Participation in the Psychosocial Health Intervention of the DC

This study analyzed data that emerged from the operation of a primary mental health center in Athens (Greece), namely, the “Day Center for the Care of the Mental Health of Women (Perinatal Mental Health Disorders)”, during the period September 2020–November 2021.This Day Center (DC), funded by the Greek Ministry of Health, was created in 2009 by FAINARETI, a Non-Profit Organization that aspires to improve perinatal care in Greece through specialized midwifery and psychosocial intervention. The Centre offers free education and counselling by midwives and antenatal/postnatal care (up to 1 year after childbirth), as well as psychological counselling and the psychiatric treatment of pregnant/new mothers and expectant/new fathers.

Pregnant women, or new mothers, and their partners can express their interest and apply for participation in the DC intervention program through a telephone intake process. An appointment is then scheduled where the woman or man are offered information on the purpose of the intervention and what it comprises. At the end of the meeting, consent to participate in the intervention is obtained. The next appointment includes a complete medical, psycho-emotional, and social-history taking by a midwife, which allows the assessment of each person’s individual needs. Based on those needs, a personalized midwifery and psychosocial intervention program is formed and is completed at the end of the 1st year postpartum.

All study participants that attended the DC’s psychosocial health intervention were offered a Midwife-led Prenatal Educational Program called “Preparation for Labor and Parenthood” (8–12 two-hour group sessions or 4–5 two-hour individual sessions). This psychosocial model is not included in regular antenatal care that is offered in Greece. The DC’s psychosocial intervention included information and counseling on the period of pregnancy, preparation for labor, breastfeeding, the needs, behavior and adjustment of the baby, baby care-taking, emotional changes for a woman/man during the postpartum period, the maternal/paternal role, and attachment development. During the program, each participant was able to attend up to 3 two-hour educational sessions pertaining to breastfeeding. Oral and written consents of all the men who participated in the Day Center’s perinatal mental health intervention program were obtained. This study was approved by the Research Ethics Committee of the Non-Profit Organization “FAINARETI” (Ref. 130/27-12-2019).

### 2.2. Study Population

This analysis includes data from all the expectant fathers who took part in the program during the period September 2020–November 2021 and accepted to participate in the study. The final sample of the study consisted of 216 men, who participated with their partners in the DC’s educational program (from approximately the 28th week of pregnancy to 12 months postpartum). In order for a man to attend the DC’s program, he must: (a) be the partner/spouse of a pregnant woman, (b) be over 18 years old, (c) not use drugs, and (d) not be in need of hospitalization due to a mental health problem.

### 2.3. Measures

The data derived from:

(A) The Medical History (general and mental health) and the socio-demographic data of men.

The history taken by a midwife before the start of the DC’s antenatal educational program (approximately between the 18th and the 22nd gestation week) included information on the men’s socio-demographic data and their mental health and well-being history (e.g., whether they received emotional support by their partners and experienced partner-relationship difficulties as well as prevalent emotions during pregnancy). This allowed the collection of necessary information for both the identification of possible risk factors in the participants’ psychosocial history and the identification of possible pathological mental-health symptoms experienced by them during their partner’s pregnancy.

(B) Psychometric Tools

The psychometric tools were administered at 2 time points: (a) 24th–28th gestation week and (b) 34th–38th gestation week, which included:

(i) Iowa Infant Feeding Attitudes Scale (IIFAS)

The IIFAS was designed by de la Mora et al. [33] to provide a reliable and valid assessment of attitudes toward infant feeding methods and to predict breastfeeding intention and exclusivity. The scale is composed of 17 items and each item is rated on a five-point Likert scale ranging from 1(strongly disagree) to 5 (strongly agree). Total scores can range from 17 to 85, with higher scores indicating a positive attitude toward breastfeeding. Total scores can be further classified into three groups: (1) positive attitude toward breastfeeding (IIFAS score 70–85), (2) neutral attitude (IIFAS score 49–69), and (3) positive attitude toward formula feeding (IIFAS score 17–48). The IIFAS has been translated and validated for Greek women and has shown high reliability (α = 0.71) [34].

(ii) Paternal Antenatal Attachment Scale (PAAS)

This scale is composed of 16 items derived from Australian samples [35]. Condon et al. [36] reviewed the results of their research on the construction and validity of parental attachment scales and found that the Father Prenatal Attachment Scale is the only scale measuring paternal attachment. It consists of two subscales: (a) the quality of attachment, which refers to the nature of the emotional experience in thinking about the fetus, and (b) the intensity of attachment, which refers to the duration of engagement with the fetus (e.g., how much time the father spends picturing the fetus in his imagination). Items are rated on a five-point Likert scale ranging from 1 to 5 [37]. Lower scores indicate less antenatal attachment. This screening tool has not been validated for the Greek population; it has only been translated for the needs of the DC’s participants. In this study, the average Cronbach alpha coefficient was 0.84.

### 2.4. Analysis

The data were analyzed using SPSS version 22.0. The description of quantitative variables was provided through mean values and the standard deviation, while absolute (n) and relative (%) frequencies were used for qualitative variables. Demographic factors included: age, education, marital status, occupation status and family financial managing. Perinatal characteristics included: perinatal education and infant feeding attitudes and paternal antenatal attachment before and after the participation in the education program. Psycho-emotional characteristics included: partner’s emotional support, relationship difficulties, prevalent emotions during pregnancy, sadness during pregnancy, and anxiety during pregnancy. In order to examine the effect of antenatal education on fathers’ attitudes toward breastfeeding and the degree of attachment to the fetus, t-test analyses were applied. Univariate analyses of variance (ANOVA) were performed to examine the relationship between socio-demographic and psycho-emotional factors and infant feeding attitudes and antenatal attachment.

## 3. Results

The demographic, perinatal and psycho-emotional characteristics of a group of 216 expectant fathers, and partners of pregnant women who participated in this study are presented in Table 1. The mean age of the men was 36.93 ± 4.17(SD) years, with almost an equal number of them having completed postgraduate (N = 84, 38.9%) and undergraduate studies (N = 81, 37.5%). More than half of the participants (N = 121, 56.0%) were private-sector employees, 31.5% of them (N = 68) were freelancers, and only 10.6% (N = 23) worked in the public sector. The majority of men were married (N = 161, 74.5%) while 23.1% (N = 50) were living with their partners with or without a cohabitation agreement. The percentage of participants who reported managing the family finances easily was 56% (N = 121).

The IIFAS and PAAS scores measured before and after the antenatal education during pregnancy are also presented in Table 1. According to the t-test analyses, the expectant fathers’ scores for infant feeding attitudes (which show breastfeeding intention and exclusivity) and prenatal attachment to the fetus were higher after participation in the antenatal education, but the difference was statistically insignificant. Regarding the expectant fathers’ attitudes toward breastfeeding, ANOVA analysis showed that none of the independent variables was associated with the men’s infant feeding attitudes (not presented in a table).

Table 2 presents the results of the univariate analyses of variance (ANOVA) for paternal antenatal attachment; only statistically significant relationships are reported. At the univariate level, the F criteria showed a statistically significant relationship among paternal antenatal attachment and six independent variables measured in the current study.

**Figure 1:**
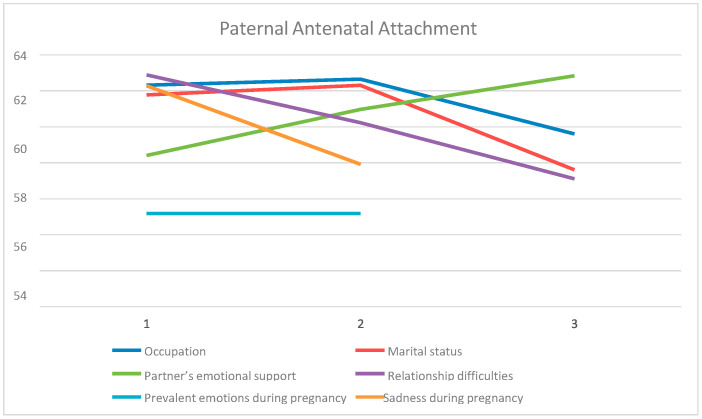
The relationship between paternal antenatal attachment and independent variables.

According to the Scheffé post-hoc criterion, in terms of occupation, there is a statistically significant difference among private-sector employees and freelancers (*p* = 0.016). Private-sector employees seemed to have greater paternal antenatal attachment, with 3.9% proportion of the variance explained by this variable. Regarding marital status, a statistically significant difference appeared among all three sub-categories (*p* = 0.026). Those with a cohabitation agreement showed greater paternal antenatal attachment than the other two groups, followed by married ones, and lastly those living together without a cohabitation agreement. The proportion of the variance explained was 3.4%. The men who felt very much supported by their partners reported greater paternal antenatal attachment compared to those with moderate or no support (*p* = 0.001), with 6.6% of the variance explained. Furthermore, the results revealed an overall tendency of significantly greater paternal antenatal attachment for the participants who had no relationship difficulties, in comparison to those who had relative and moderate difficulties. Between the last two groups, those with relative difficulties showed higher prenatal attachment (*p* < 0.001). The proportion of the variance explained was 8.0%. The largest proportion of the variance (20.9%) in the current study was explained by prevalent emotions during pregnancy, where those being very happy during pregnancy had greater paternal antenatal attachment than those who reported being ‘just’ happy (*p* < 0.001). None of the participants expressed negative prevalent emotions. Finally, a 4.3% proportion of the variance was explained by the independent variable of sadness during pregnancy (*p* = 0.009). Analysis showed that those with no such emotion experienced greater paternal antenatal attachment in relation to those with a small to moderate presence of sadness (Table 2). Figure 1 shows the relationship of the means of paternal antenatal attachment according to the independent variables after univariate analysis (ANOVA).

## 4. Discussion

The purpose of this study was not only to investigate the impact of antenatal education on expectant fathers’ attitudes toward breastfeeding and attachment to the fetus, but to also explore psycho-emotional characteristics which are associated with these issues. Even though the expectant fathers’ scores about breastfeeding intention and exclusivity were higher after their participation in the antenatal education program, the difference was statistically insignificant. These findings are in contrast to those of Raeisi et al. [38] who found that antenatal education can influence men’s attitudes toward breastfeeding. However, it is important to keep in mind that the sample of expectant fathers in this study had expressed an interest in participating in antenatal education in order to acquire information on breastfeeding and be better prepared. This shows that they were already positively inclined toward breastfeeding and may explain the specific findings of this study.

In addition, demographic factors such as educational level, marital status, financial level, and occupation of the expectant fathers did not appear to correlate with their attitude toward breastfeeding in this study. On the contrary, in studies by Pollock et al. [39], Vaaler et al. [40] and Chezem [41], it was shown that fathers’ attitudes were associated with ethnicity, cultural background and educational level. More specifically, students showed lower scores on scales assessing their attitudes toward breastfeeding, both in comparison to employed and unemployed men. Older men showed higher scores on this scale. The absence of a correlation between men’s attitudes toward breastfeeding and their educational level has been shown, apart from this study, in the research of Laanterä et al. [42].

The international literature has indirectly highlighted the value of antenatal education through the association between knowledge and attitude. Lack of knowledge appears to be responsible for misconceptions toward breastfeeding [39], while positive attitudes toward breastfeeding appear to be associated with higher levels of knowledge [43]. Freed et al. [44] found similar relationships between attitudes and knowledge among men in the USA (n = 268). Men with positive attitudes toward breastfeeding believed that it was better for the infant, that it helped to a greater extent with mother–infant bonding, and that it protected the infant against illness. Addressing myths and misconceptions antenatally can be instrumental in overcoming barriers to breastfeeding initiation and providing greater family support to breastfeeding mothers [44]. Therefore, partner involvement in breastfeeding-related discussions and education, both in the context of antenatal visits to health professionals and participation in preparation classes, should always be encouraged [39].

Although this study did not identify any factors that could positively influence men’s attitudes toward breastfeeding and considering the value of the partner’s role during breastfeeding, further research on additional factors is needed in the future. For example, previous studies have shown an association between fathers’ attitudes toward breastfeeding and whether they had been breastfed as children [39,42]. It is also possible that further investigation on expectant fathers’ attitudes toward breastfeeding and antenatal education will reveal more encouraging results. It may be that men’s participation in such antenatal groups could positively influence their attitudes toward breastfeeding and potentially increase breastfeeding rates in a country.

Regarding the expectant fathers’ attachment to the fetus, this study showed that fathers had higher rates of antenatal attachment after the completion of the antenatal program compared to its start, yet the difference did not appear to be statistically significant. Serçekuş et al. [45] also found that antenatal education has no influence on maternal and paternal attachment. In contrast, a relationship between fathers’ antenatal education and antenatal attachment to the fetus has been shown in a recent study by Setodeh et al. [46]. However, this result may be explained by the fact that in the study by Setodeh et al. [46], the education program was more aimed at the development of the degree of attachment to the fetus. A similar positive correlation was shown in the very recent study by Türkmen et al. [28], but no details were given on the type and number of antenatal meetings that preceded the study.

The finding that men who reported receiving a lot of emotional support from their partner and experiencing no difficulties in their relationship showing higher rates of fetal attachment is consistent with results of other studies that indicated a positive correlation between antenatal attachment and the quality of the partner relationship [47,48]. International literature has identified the quality of the partner relationship, as well as the support and intimacy between partners, as the most important factors in terms of the degree of paternal attachment to the fetus [49,50,51].

Furthermore, this study did not show a correlation between desire for the pregnancy and the development of a bond with the fetus antenatally. Given that attachment in this study was measured after the middle of pregnancy and considering that the degree of attachment increases with gestational age [37,52,53], the finding that men developed prenatal attachment to the fetus even in cases where the pregnancy was not intended seems reasonable.

According to the international literature, there are conflicting results regarding the factors that are both associated with and which influence the development of antenatal attachment between the expectant father and the fetus. For example, when it comes to the father’s age, Tolman et al. [54] and Ustunsoz et al. [55] argue that the relationship is inversely proportional, i.e., younger men report higher levels of attachment to the fetus and future child than older men. In contrast, other studies [28,46] have indicated that older men (>35 years old) show higher reported attachment. In this study, no correlation between the age of the expectant father and the development of attachment to the fetus emerges. Recent research has identified other factors that appear to have a positive association with antenatal father–fetus attachment, but these factors were not examined in this study. Such factors include the gender of the child, the relationship of participants in antenatal education groups with their parents, and whether the participants have other children [28].

There have been many studies published in the literature on postnatal maternal–fetal and paternal–fetal attachment. However, studies on fathers’ attachment to the fetus during pregnancy are limited [37,49,50,56,57,58]. It is already known from the literature that the higher the paternal–fetal attachment is during pregnancy, the higher it is postnatally [49], and that men with higher paternal–fetal attachment take on more responsibilities as fathers and interact consistently and qualitatively with their children [46]. Therefore, it is especially important to determine the factors that negatively affect expectant fathers’ attachment to the fetus. Determining these factors and designing interventions that aim to improve attachment would increase paternal–fetal attachment [46]. Moreover, further investigation on the impact of expectant fathers’ antenatal education on antenatal attachment to the fetus may reveal additional encouraging results.

To our knowledge, this is the first study to simultaneously investigate expectant fathers’ attitudes toward breastfeeding and antenatal attachment to the fetus. One limitation is that the sample in this study largely consisted of university-educated men with satisfactory or high incomes. Furthermore, this research was conducted in a specific primary mental health care setting and the participants came mainly from the region of Attica (with a few exceptions). Therefore, the sample is not considered to be strictly representative of the Greek expectant fathers’ population and the findings of the study may not be generalizable. Another limitation of this study may be its focus on men expecting their first child. The course of development of paternal–fetal attachment may be similar or different for fathers with previous pregnancy experience. In addition, the sample comprised expectant fathers who had already expressed interest in participating in childbirth and parenting preparation education during pregnancy. It should, therefore, be borne in mind that all men in this sample were personally motivated and willing to be informed and educated on breastfeeding and attachment to the baby. An additional limitation concerns the scoring parameters used for the Paternal Antenatal Attachment Scale (PAAS) given that it is not yet validated for the Greek population. A final limitation is the fact that the psychometric tool scores were obtained after the middle of pregnancy onwards rather than at the beginning. However, it is important to mention that in Greece, as in other countries internationally [59,60], antenatal education for pregnant women and their partners is provided after the 20th gestation week and not since the beginning of pregnancy.

## 5. Conclusions

In this study, the expectant fathers’ scores, which show breastfeeding intention/exclusivity and prenatal attachment to the fetus, were higher after their participation in the antenatal education program performed by midwives, compared to their scores at the beginning of the program, although the difference was statistically insignificant. Also, this study presents some factors, such as the emotional support from partners and the absence of relationship difficulties that were associated with increased rates of paternal antenatal attachment to the fetus. Given the value of the fathers’ role during breastfeeding, future research should be directed toward the identification of factors that impact the expectant fathers’ attitudes toward breastfeeding. In addition, further research is needed to determine additional factors that may affect paternal antenatal attachment during pregnancy.

## Figures and Tables

**Table 1:** Expectant Fathers’ Demographic, Perinatal and Psycho-emotional Characteristics.

Demographic Characteristics	*N* *M*	*%* *SD*
Age	36.93	4.17
Education		
High school	51	23.6
Bachelor’s degree	81	37.5
Postgraduate studies	84	38.9
Total	216	100.0
Occupation		
Private-sector employee	121	56.0
Public-sector employee	23	10.6
Freelancer	68	31.5
Total/Missing	212/4	98.1/1.9
Family financial managing		
Sometimes difficult	44	20.4
Not that well	50	23.1
Easy	121	56.0
Total/Missing	215/1	99.5/.5
Marital status		
Married	161	74.5
Cohabitation agreement	32	14.8
Living with partner	18	8.3
Total/Missing	211/5	97.7/2.3
*Perinatal Characteristics*		
Antenatal education		
No	31	14.29
Yes	185	85.71
Total	216	100.0
IIFAS score at the beginning of the program	63.98	7.86
IIFAS score at the end of the program	65.48	8.55
PAAS score at the beginning of the program	61.46	6.57
PAAS score at the end of the program	64.99	7.16
*Psycho-emotional Characteristics*		
Partner’s emotional support		
Not at all to Moderate	41	19.0
Quite a lot	62	28.7
Very much	113	52.3
Total	216	100.0
Relationship difficulties		
No difficulties	124	57.4
Relative difficulties	72	33.3
Moderate difficulties	20	9.3
Total	216	100.0
Prevalent emotions during pregnancy		
Happy	25	11.69
Very happy	191	88.31
Total	216	100
Sadness during pregnancy		
Not at all	163	75.32
A bit to Moderate	53	24.68
Total	216	100
Anxiety during pregnancy		
Moderate	34	15.58
Quite a lot	80	37.16
Very much	102	47.26
Total	216	100

*Note. M*—mean; *SD*—standard deviation; *N*—frequencies; %—relative frequencies.

**Table 2:** Univariate Analyses of Variance of Paternal Antenatal Attachment.

Paternal Antenatal Attachment						
	*M*	*SD*	*F*	*df*	*p*	*η* ^2^
Occupation						
Private-sector employee	62.32 a	5.95				
Public-sector employee	62.65 ab	4.90	4.189	2	0.016	0.039
Freelancer	59.60 b	7.85				
Marital status						
Married	61.78 a	6.56				
Cohabitation agreement	62.31 b	6.17	3.699	2	0.026	0.034
Living with partner	57.61 c	5.65				
Partner’s emotional support						
Not at all to Moderate	58.41 a	9.69				
Quite much	60.97 ab	5.01	7.494	2	0.001	0.066
Very much	62.84 b	5.50				
Relationship difficulties						
No difficulties	62.89 a	5.479	9.259	2	*p* < 0.001	0.080
Relative difficulties	60.229 b	7.09				
Moderate difficulties	57.109 c	8.279				
Prevalent emotions during pregnancy						
Happy	55.18	8.21	56.523	1	*p* < 0.001	0.209
Very happy	62.89	5.19				
Sadness during pregnancy						
Not at all	62.28	6.53	4.789	1	0.009	0.043
A bit to Moderate	57.92	6.39				

*Note. M*—Mean; *SD*—Standard Deviation, *F*- F Criterion, *df*—Degrees of freedom, *p*—Statistical Significance, *η*^2^—eta-Squared index; Means that share a common index (a, b, c) do not differ significantly from each other according to the Scheffé post-hoc criterion.

## Data Availability

Data are contained within the article.
